# Slow sand filtration of raw wastewater using biochar as an alternative filtration media

**DOI:** 10.1038/s41598-020-57981-0

**Published:** 2020-01-27

**Authors:** Korbinian Kaetzl, Manfred Lübken, Edith Nettmann, Stefan Krimmler, Marc Wichern

**Affiliations:** Institute of Urban Water Management and Environmental Engineering, Ruhr-Universität Bochum, Fakultät für Bau- und Umweltingenieurwissenschaften, Universitätsstr. 150, 44780 Bochum, Germany

**Keywords:** Environmental microbiology, Ecology, Environmental sciences

## Abstract

The efficiency of anaerobic biofilters (AnBF) as low-cost wastewater treatment systems was investigated. *Miscanthus*-biochar was used as filtration media and compared with sand as a common reference material. Raw sewage from a municipal wastewater treatment plant was stored in a sedimentation tank for two days to allow pre-settlement of wastewater particles. Subsequently, wastewater was treated by AnBFs at 22 °C room temperature at a hydraulic loading rate of 0.05 m∙h^−1^ with an empty bed contact time of 14.4 h and a mean organic loading rate of 509 ± 173 g_COD_∙m^−3^∙d^−1^. Mean removal of chemical oxygen demand (COD) of biochar filters was with 74 ± 18% significantly higher than of sand filters (61 ± 12%). In contrast to sand filters with a mean reduction of 1.18 ± 0.31 log-units, *E*. *coli* removal through biochar was with 1.35 ± 0.27 log-units significantly higher and increased with experimental time. Main removal took place within the *schmutzdecke*, a biologically active dirt layer that develops simultaneously on the surface of filter beds. Since the *E*. *coli* contamination of both filter materials was equal, the higher removal efficiency of biochar filters is probably a result of an improved biodegradation within deeper zones of the filter bed. Overall, performance of biochar filters was better or equal compared to sand and have thus demonstrated the suitability of *Miscanthus*-biochar as filter media for wastewater treatment.

## Introduction

In many fast developing economies, the agricultural sector accounts for up to 90% of the fresh water withdrawals^1^. At the same time only 10% of the generated wastewater in these countries undergoes an appropriate treatment^2^. The release of untreated or partially treated wastewater into the environment causes serious impairments with enteric microorganisms, as such as *Escherichia coli* and organic compounds with potential risks for irrigation and drinking water sources^3^. Hence, there is an urgent need to develop a simple and efficient wastewater treatment system with locally available resources. A widespread implementation in low income countries will contribute to a substantial reduction of the associated health risks.

Slow sand filtration (SSF) is generally considered to be one of the most efficient and at the same time very favorable technology for the reduction of pathogens, particulate organic substances and turbidity^4–6^. Its suitability as tertiary treatment option for secondary effluents on municipal wastewater treatment plants (WWTPs) to reach an appropriate quality for safe irrigation water with a relatively high hydraulic loading rate (HLR) of more than 0.05 m∙h^−1^ was confirmed by several studies. For instance, Langenbach *et al*.^7^ reported an average reduction of fecal indicator bacteria (FIB) of 1.95 to 2.46 log-units, while Pfannes *et al*.^8^ found an elimination between 1.58 and 2.26 log-units. For coliforms, a reduction of 1.4 to 2.0 log-units was reported by Ellis^9^ and for total coliforms a removal between 0.68 and 2.0 log-units^10,11^. However, SSF requires sand with specific properties regarding grain size diameter and uniformity coefficient, which might be not locally available in some regions^12,13^.

In this context, biochar was successfully tested as an additive in wastewater treatment systems^14^. The suitability of biochar as low-cost sorbent for the removal of metal ions, ammonium and phosphate from water and wastewater was investigated in several studies^15–17^. Dalahmeh *et al*.^18^ used biochar filters as an on-site sewage treatment for the removal of pharmaceuticals from wastewater and found, particularly due to adsorption of persistent substances, a significant higher elimination of the investigated substances in biochar columns in comparison to the tested sand filters. Perez-Mercado *et al*.^19^ investigated the suitability of biochar filters for wastewater treatment and reported higher total nitrogen removal for biochar filters compared to sand filters. Mohanty and Boehm^20^ treated stormwater with biochar augmented sand biofilters and found an improved retention of *E*. *coli* (~96%) in biochar columns compared to sand (~35%) and explained this result by higher pathogen adsorption rates on biochar particles.

However, there is still a significant knowledge gap regarding the suitability of SSF as anaerobic treatment of wastewater and the reduction of fecal indicator bacteria (FIB)^21^. Also insights in the efficiency of biochar as an alternative filter material to sand for anaerobic slow sand filtration, further designated as anaerobic biofiltration (AnBF), are scarce. By using mostly artificial wastewater, current research focuses either on the adsorption of FIB on biochar particles during shock loads or on the degradation under aerobic conditions. In addition, most of the experiments with biochar for wastewater treatment have so far been carried out at very low organic loading rates and with relatively long hydraulic residence times. The resulting large area requirement for such wastewater treatment systems, however, makes practical implementation more difficult. To close knowledge gaps, the aims of this study were (i) to investigate the applicability of AnBF for raw wastewater treatment with respect to the reduction of (ii) the FIB *E*. *coli* and enterococci and (iii) physico-chemical parameters, as such as chemical oxygen demand (COD), total organic carbon (TOC), ammonia nitrogen (NH_4_-N) and total phosphorous (P_tot_). Furthermore, (iv) the suitability of *Miscanthus*-biochar as filter material was evaluated and compared with sand as common reference material.

## Results and Discussion

### Physico-chemical parameter

**Figure 1:**
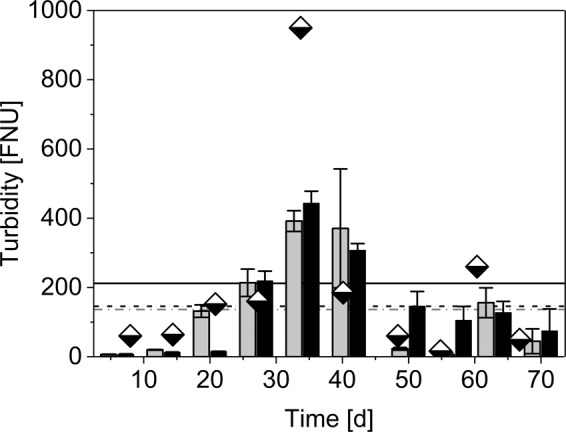
Mean effluent turbidity of sand filters (grey) and biochar filters (black) over experimental time. Error bars represent standard deviations. Influent turbidity is marked as half-filled diamonds. Horizontal black solid line represents mean influent turbidity, black dashed-dotted line represents mean effluent turbidity of biochar filters and grey dashed-dotted line mean effluent turbidity of sand filters.

Physico-chemical parameters of inflow were unstable over experimental time (Table 1). A significant difference was found for turbidity in filter influent and effluent, as well as between sand and biochar columns (Fig. 1). Noticeable were the low turbidity values (>20 FNU) for filter effluents on the first two sampling days. During the third week, a breakthrough of turbidity was observed for sand filters and one week later for biochar filters. From this breakthrough on, effluent turbidity was highly unstable and exceeded occasionally similar values as measured for the influent, particularly at the halftime of the experiment. Neither a correlation between inflow and outflow, nor a significant difference between the filter materials could be observed apart from the start-up phase. The later turbidity breakthrough of biochar filters might be explained by a higher particle retention and adsorption capacity of biochar due to their notable higher specific surface area (Table S1)^14^.

**Table 1:** Mean influent and effluent concentrations of different filter materials over experimental time.

Parameter	Unit	Influent	Effluent	
			Sand	Biochar
Chemical oxygen demand (COD)	mg∙L^−1^	305^a^ ± 104 (4)	116^b^ ± 52 (12)	69^c^ ± 26 (12)
Total organic carbon (TOC)	mg∙L^−1^	93^a^ ± 31 (4)	50^b^ ± 18 (12)	34^c^ ± 8.8 (12)
Ammonia nitrogen (NH_4_-N)	mg∙L^−1^	42^a^ ± 19 (4)	40^a^ ± 9.6 (12)	39^a^ ± 9.3 (12)
Total phosphorous (P_tot_)	mg∙L^−1^	6.5^a^ ± 0.6 (4)	4.9^b^ ± 1.3 (12)	4.3^b^ ± 0.4 (12)
Total suspended solids (TSS)	mg∙L^−1^	57^a^ ± 12 (4)	NA	NA
Electrical conductivity (EC)	µS∙cm^−1^	1077^a^ ± 387 (9)	1040^a^ ± 402 (24)	1130^a^ ± 308 (24)
Turbidity	FNU	212^a^ ± 287 (9)	136^a^ ± 150 (24)	146^a^ ± 140 (24)
pH		7.4^a^ ± 0.2 (9)	7.6^a^ ± 0.3 (24)	7.6^a^ ± 0.5 (24)
*E*. *coli*	log_10_MPN∙100 mL^−1^	6.2^a^ ± 0.65 (9)	4.9^b^ ± 0.7 (24)	4.8^c^ ± 0.5 (24)
Enterococci	log_10_MPN∙100 mL^−1^	5.5^a^ ± 0.69 (9)	NA	NA

Standard deviations are presented as variation range. Letters indicate a significant difference of the mean (*p* < 0.05) after post-hoc analysis. Number of samples in brackets. *E*. *coli* and enterococci concentrations are given in Most Probable Number. Number of samples in brackets.

Despite the relatively large grain size of filter materials, it can be expected that the construction of the filter columns supported particle removal through both surface filtration and deep bed filtration^22^. However, the turbidity breakthrough might be a result of an organic overload (Table 1) that exceeded the retention capacity of the filter beds. This is supported by the correlated normalized turbidity and COD concentrations of both filter materials (Fig. S7c,d). The high nutrient availability in raw wastewater supported the biomass growth in the filter beds and seems to have led to a wash-out of excess biomass. Such wash-out process was evident in the high turbidity rates. These findings are in line with outcomes from Ari and Adin^23^, who treated primary and secondary wastewater effluents with SSF with an effective grain size of 0.64 mm and a HLR of 0.15 m∙h^−1^ and a high organic load. They also determined an increase in effluent turbidity after three weeks due to a high biological activity as well as notably higher values for filter effluent than in the raw water.

Both filter materials removed a substantial amount of COD and TOC from raw wastewater. Average COD and TOC removal in biochar columns was with 74 ± 18% and 61 ± 12% significantly higher than in sand filters (61 ± 12% for COD and 46 ± 3.8% for TOC). Analogous to the turbidity, COD removal of sand filters declined after two weeks from 70% to 50% and from more than 80% to 50% for biochar filters after three weeks. Towards the end of the experiment, COD removal rates stabilized between 65–75%, with higher values for biochar filters. The decreasing COD removal can be explained by a release of excess biomass and partly degraded organic particles, as described above. The increase in materials’ removal efficiency towards the end of the experiment indicates an increased biological activity and thus a more efficient biological degradation of organic matter in deeper filter zones as well as an improved particle retention in the developed *schmutzdecke* on the filter surface. Higher removal efficiency of COD and TOC in biochar columns can be explained by positive effects of biochar on anaerobic degradation processes, such as electron transfer, improved colonialization and buffering capacity or adsorption of inhibitors^24,25^.

While NH_4_-N was not significantly affected by the filtration, probably due to the reduction of organic nitrogen under anaerobic conditions, phosphorous (P_tot_) removal was with roughly 35% significantly higher in biochar columns than in sand filters (around 25%). The high P_tot_ removal through both filter materials cannot be explained solely by the incorporation into the biomass^26^. Hence, a phosphorous removal through surface filtration of organic compounds, as well as by precipitation of phosphate ions in the filter bed by present cations and oxides (e.g. magnesium oxides; MgO) can be assumed^27^. The higher removal rates of phosphorous in biochar filters can be explained by the high adsorption potential of biochar as reported elsewhere^16,28^.

Correlation analyses between physico-chemical parameters over experimental time revealed a more pronounced collinearity between effluent concentration, normalized concentration (C⋅C_0_^−1^) and removal efficiency in biochar filters compared to sand filters (Fig. S7). However, a very strong relationship between COD and TOC was observed in both filter materials. Considering normalized concentrations, also COD and turbidity were strongly correlated (positively) in both filter materials. In biochar filters, the normalized concentration of all chemical parameters increased significantly over experimental time (Fig. S7c) and was related to a decrease in the removal efficiency of these parameters (Fig. S7e). As such, correlation analyses support our hypothesis of unstable and high turbidity values in filter effluent as a result of a biomass wash-out.

Nevertheless, the duration of the experiment and the number of samples do not allow a full clarification of the interaction of processes which were involved in the removal of chemical parameters and organic matter from raw wastewater through AnBF.

### Filter run time and clogging

**Figure 2:**
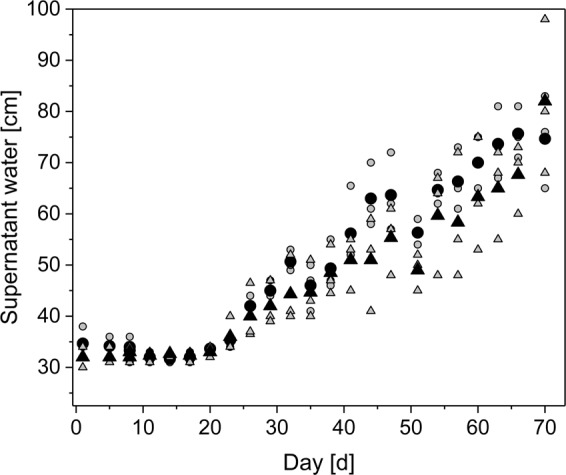
Development of the supernatant water level over experimental time for sand and biochar filters. Sand filters are presented as circles (mean values as black circle (●) and each single filter as grey circle ()) and biochar filters as triangles (mean values as black triangle (▲) and each single filter as grey triangle ()).

To compensate high TSS and COD concentrations in the untreated wastewater, filter materials with relatively large grain sizes were chosen to avoid rapid filter clogging (Fig. S1). Although grain size distribution and porosity of biochar and sand differed, the behavior of the supernatant water layer was similar for both materials (Fig. 2). The supernatant water was constant at adjusted 30 cm for the first 20 days. Subsequently, the water layer rose linearly until a water overflow occurred after 70 days for one filter. This indicates that the development of a *schmutzdecke* and the associated filter clogging processes were similar for both materials. Thereby, the achieved filter run time of 70 days was comparable to Farooq and Al-Yousef^10^ who reported an operational run of 84 days, treating secondary effluent with SSF with an effective size of 0.56 mm and a HLR of 0.16 m∙h^−1^. However, other studies reported a significant longer operation time treating secondary clarifier effluent with SSF^7^.

After 70 days, the upper 10 cm of each filter was removed and replaced by new material to reduce the filter resistance through biofilm covering, as known from slow sand filtration processes^7,12^. However, due to an intensive accumulation of biomass and organic matter within deeper filter zones, as a result of slow anaerobic hydrolysis and degradation^26^, this treatment was not sufficient in preventing filter overflow that occurred already after one week within both filter types. This indicates that the penetration of the *schmutzdecke* and filter clogging in AnBFs take place at deeper filter zones compared to SSF filtering surface water or secondary clarifier effluent^7,9,10,29^ and is caused by large grain size of filter material and high OLR^12,29,30^. Since the construction of the filter columns did not permit backwashing to remove physical blockages without compromising the filter bed^12^, larger parts of the filter bed must be replaced to prolong filter runtimes. However, to prevent a fast increase of the supernatant water layer and filter clogging in the upper part of the filter bed and thus to extend filter lifetimes, a protection layer on the filter surface can be added^31^.

### Microbiological parameter

Over the entire experiment, influent FIB concentration varied strongly, and the concentration of *E*. *coli* ranged from 4.9 to 6.9 log_10_MPN∙100 mL^−1^ (mean: 6.2 ± 0.65) and from 4.4 to 6.5 log_10_MPN∙100 mL^−1^ (mean: 5.5 ± 0.69) for enterococci (Table 1). The average *E*. *coli* removal in biochar filters was with 1.35 ± 0.27 log_10_MPN∙100 mL^−1^ significantly higher compared to sand filters (1.18 ± 0.31 log_10_MPN∙100 mL^−1^; t-test, *p* < 0.05; Table 1). Maximum reduction of *E*. *coli* could be achieved with 1.9 log_10_-units through biochar filters and 1.7 log_10_-units through sand filters. As filtration process led to the formation of an inhibitor that affected test procedure for enterococci, reduction of enterococci, however, could not be determined. Over the entire experiment, biochar filters showed a slightly more constant and higher removal efficiency than sand filters with a difference of up to 0.5 log units at the end of the experiment (Fig. S3).

Because of different operation parameters, as such as grain size, filter bed depth, raw water quality and HLR, a comparison of our results with those of similar studies can only be done cautiously. Nevertheless, similar removal rates of *E*. *coli*, which were achieved in this experiment, were reported i.a. by Ellis^9^ who treated secondary clarifier effluent with SSF with a HLR of 3.5 m∙d^−1^ and a sand bed with an effective diameter of 0.6 mm (U: 1.2) and achieved an average reduction of coliforms of 97% (~1.5 log-units). Bellamy *et al*.^32^ reported an elimination of total coliforms of 85% (~0.8 log-units) during the startup phase of a new sand filter treating surface water. Farooq and Al-Yousef^10^ reported a total coliform reduction of 93% (~1.2 log-units) for treating secondary effluent with a sand bed depth of 55 cm.

**Figure 3:**
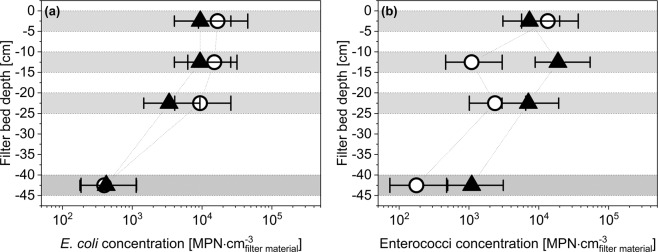
Contamination of filter material with *E*. *coli* (**a**) and enterococci (**b**) for sand (circles) and biochar (black triangle) filters. Error bars represent 95% confidence interval. Horizontal grey bars represent sampling zones in the filter bed.

In our study, biochar filters’ removal efficiency of *E*. *coli* increased over experimental time (*R* = 0.81, *p* < 0.01) and was expressed through a decrease in the normalized concentration of *E*. *coli* (Fig. S7). In contrast, the removal efficiency of sand filters was decreasing over experimental time and was expressed by an increase in the normalized *E*. *coli* concentration. Regarding the removal mechanisms of *E*.*coli* in AnBFs, the sampling at different filter depths revealed that the *schmutzdecke* was with more than 1 log_10_-unit mainly responsible for the removal of pathogens (Fig. S2). Thus physical surface filtration and subsequently biodegradation within the *schmutzdecke* can be considered as the main removal mechanisms in AnBFs, as confirmed by other studies^7,9,29^ and explains the relatively high removal rate shortly after filter activation with still clean filter material. Especially sand filters were shown to have limited removal capacities within deeper filter layers. In contrast, the constructed biochar filter seemed to be able to remove pathogens also within deeper filter zones as between the first sampling port below the *schmutzdecke* and the filter outflow an additional removal of nearly 0.5 log_10_-units was detected. Since the formation of the *schmutzdecke* was comparable for both materials and the FIB contamination in a similar range (Fig. 3), the increasing removal efficiency of biochar filters cannot be solely explained through an improved surface filtration or adsorption. The likely causes for an improved removal of *E*. *coli* through biochar filters seems to be, firstly, the increased biological activity and, secondly, an additional degradation within deeper zones of the filter.

### Filter material

The used biochar was characterized by a low H/C-(0.19) and O/C-(0.08) ratio (Table S2), indicating a high degree of carbonization with a very strongly condensed aromatic ring system and a low number of functional groups^33^. Hence, a high chemical stability against microbial degradation can be expected from biochar and qualifies it as a suitable filtration media for AnBF of wastewater^12,34^. The used quartz sand in turn did not contain chemical impurities (Table S3).

In terms of the physical properties, biochar has a significantly higher specific surface area compared to sand (Table S1). As such, biochar provides various habitats for microbes for colonization and growth, and where they are protected from being grazed by predators^35^. However, the high surface area of biochar results mainly from micropores (<1 nm), which do not contribute to effectively adsorption of colloids, such as FIB, from aqueous solutions.

To determine the effect of filter materials on FIB adsorption and biofilm formation, samples from different depths were taken at the end of the experiment. Samples were analyzed regarding their contamination with FIB as well as for biofilm covering using confocal-laser scanning microscope (cLSM) and scanning electron microscope (SEM) images. The measured FIB contamination for *E*. *coli* was in the range of 4∙10^3^–1.7∙10^4^ MPN∙cm^−3^ and for enterococci between 1.8∙10^2^–1.9∙10^4^ MPN∙cm^−3^ (Fig. 3). The pollution with FIB decreased with filter bed depth and was lowest at the bottom of the filters. The relatively small decline of the upper 25 cm of the filter bed can be explained by the high biomass load, which led to filter clogging in deeper zones as discussed above. Due to their porous structure, biochar was expected to be more contaminated with FIB, but the measured differences between the materials were not significant. An improved retention of FIB in biochar or biochar-augmented filters was reported by other studies^20^ and was explained by the higher adsorption capacity of biochar compared to sand. However, the outcomes of this study cannot confirm this hypothesis.

**Figure 4:**
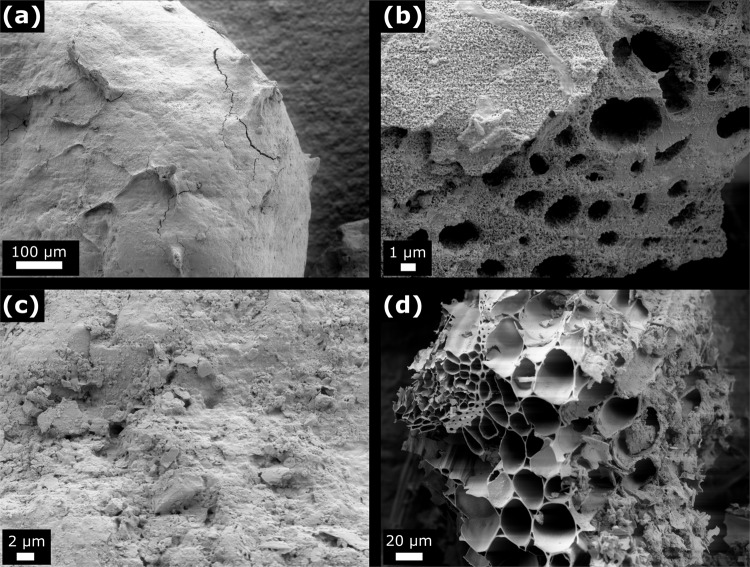
Scanning electron images of filter material after 70 days. At 10 ± 2.5 cm filter depth of sand (**a**) and biochar (**b**) and at 30 ± 2.5 cm filter depth of sand (**c**) and biochar (**d**).

Additional samples from filter material at 10 ± 2.5 cm and 30 ± 2.5 cm (Fig. 4, Figs. S4 and S5) were analyzed with scanning electron images for biofilm development and presence over filter bed depth. Like the measured FIB contamination, a decrease of biofilm covering with filter depth could be observed. At 10 cm depth, the material was fully covered with biofilm structures and even within the macroscopic pores of biochar, biofilm formation could be observed. In contrast to this massive biomass growth, only a partly and thin biofilm development could be detected in deeper zones.

**Figure 5:**
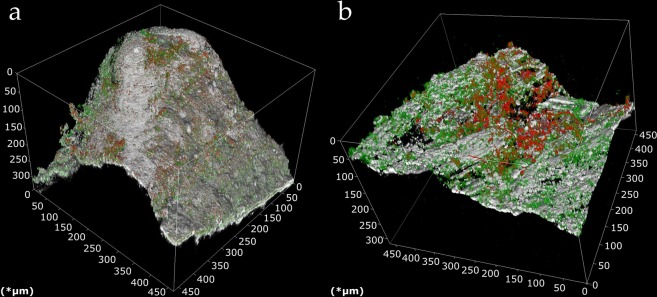
3D confocal laser scanning micrographs from sand (**a**) and biochar (**b**) particles. Images show total bacteria (red), EPS-Glycoprotein (green), and reflection of particle surface (grey).

Since SEM images were not preserving the biofilm structure, as samples had to be dehydrated before analysis, cLSM images were taken to investigate biofilm on particles *in situ* (Fig. 5). Images were analyzed with respect to total bacteria cell number, extracellular polymeric substances (EPS) and stack volume (Fig. S6). Thereby, no significant differences could be found for biochar and sand particles. However, due to the porous and fractal structure of biochar particle, the laser was not able to penetrate all pores and areas, wherefore the detected signals possibly underestimated the real EPS and cell number on biochar particles (Fig. S6). Accordingly, no significant effect of the assumed higher adsorption capacity of bacteria on biochar could be observed over the whole experimental period of 70 days. Due to the high OLR of the AnBFs, which was notably higher than in comparable studies^7,8,18,19^, it can be assumed that the adsorption capacity of biochar was exhausted in an early stage of our experiment. This is supported by the fact that the main removal of *E*. *coli* in the AnBFs took place within the *schmutzdecke*. Hence, adsorption of FIB on the filter bed can be expected as a minor contribution to the overall removal from wastewater.

As soon as the filter lifetime is reached and filters need to be dismantled, the used filter material can be recycled. However, reuse strategies for sand are limited. Nevertheless, sand has the potential to be reused as filtration media after washing and removing of biomass. Also, a recycling as construction material (e.g. for concrete) might be possible.

In contrast to sand, biochar can be used for CO_2_ neutral energy production or cooking by combustion^36^. Also a hygienization through co-composting might be a potential strategy^37^, which in turn could reduce the greenhouse gas emissions of composting^38^. After hygienization, biochar material can be used as soil amendment^39^. Also a reactivation of the biochar by pyrolysis or the production of activated carbon by steam activation might be possible recycling strategies. And due to positive effects on methane yield, the utilization in biogas plants is further conceivable^24,25^.

### General discussion

The organic loading rate of filters was too high to achieve a sufficient operation time. Hence, either the HLR needs to be reduced or an improved pre-treatment of municipal wastewater must be developed to increase the operation time of AnBF and removal efficiency.

A lower HLR would result in a longer HRT and in a lower OLR and thus in a more efficient inactivation rate of FIB^40^. On the other side, a longer HRT would result in a larger area demand of the wastewater treatment plant, which might be a limiting factor in regions where available areas are restricted.

Furthermore, as hydrolysis of organic substances is assumed to limit anaerobic degradation processes^41^, a lowering of OLR would lead to a reduced accumulation of organic matter. With an improved wastewater pre-treatment, such as pond systems or anaerobic filters to remove organic substances^9,42,43^, longer filter run times can be expected of AnBFs by preserving the relatively high HLR. This in turn may improve the formation of a well-developed biofilm in the filter bed, which is expected to increase the removal efficiency of pathogens and organic substances.

Higher removal rates of *E*. *coli*, COD and TOC through biochar filters indicates enhanced microbial decomposition during the filtration. However, cLSM microscope analysis showed no significant differences in the abundance of total bacteria (Fig. S6). Hence, further research must be conducted regarding the microbial composition of the biofilm on biochar in AnBFs for wastewater treatment.

## Conclusion

Our results could show that AnBFs constitute a suitable treatment method to reduce organic compounds and FIB from pre-settled raw wastewater. Biochar filter performance was comparable to that of the commonly used sand filters and for some parameters, biochar filters even exceeded the performance of sand filters. Biochar, as in our case biochar from *Miscanthus* grass, is a highly promising filter material for AnBF, particularly since biochar can be produced locally from agricultural residues with recycling opportunities for energy production or soil amelioration after its use as filter material for wastewater treatment. AnBF with biochar as filter material might be also an effective short-term wastewater treatment in emergency situations. However, to achieve a water quality that fulfills for instance the WHO guidelines for unrestricted irrigation, further research is needed that aims to achieve longer filter run times and higher and more constant removal rates of FIB.

## Material and Methods

### Biofilter materials

Two different materials, namely quartz sand and *Miscanthus* biochar, were tested for their suitability as filter materials for anaerobic wastewater treatment using anaerobic vertical downflow biofilters (AnBFs). Both filter material consisted of a relatively large grain size (Table S1; Fig. S1), which is commonly used for rapid filtration^12^. However, for our set up larger grain size of filter material was necessary to achieve longer filter run times and prevent a rapid filter clogging^30,44,45^, as the used raw wastewater consisted of a high concentration of suspended solids (Table 1). Nevertheless, according to Unger and Collins^46^, the used grain size is suitable to achieve adequate removal rates in SSF.

Quartz sand (effective diameter (d_10_): 0.86 mm; uniformity coefficient (U): 1.47) was used as common reference material. *Miscanthus* biochar (d_10_: 0.58 mm; U: 3.12) was produced in a pyrolysis reactor at 850 ± 20 °C with a residence time of 30 min (Pyreg GmbH, Doerth, Germany). According to the manufacturer, the used biochar meets the requirements of the European Biochar Certificate^33,47^. Before being used, biochar was sieved over a 1 mm sieve to separate fine particles. Filter material was characterized with respect to specific surface area, porosity, grain size distribution, HRT as well as for chemical composition (Tables S1–S3). The surface area of biochar (500 m²∙g^−1^) was determined according to DIN ISO 9277, while the surface area of sand (<0.01 m²∙g^−1^) was calculated assuming a spherical shape of the particles according to Wichern *et al*.^48^. Elemental composition of biochar (C, N, H, O) was measured in triplicates according to DIN 51732, using a Vario EL Cube elemental analyzer (Elementar Analysensysteme GmbH, Langenselbold, Germany). Ash content was measured according to ASTM D1762-84^49^ and main elements (P, Al, Fe, K, Mg, Na) in biochar were measured after microwave digestion with nitric acid in teflon tubes and subsequently elements were determined with an ICP-OES (Ciros CCD, SPECTRO Analytical Instruments GmbH, Kleve, Germany). Chemical composition of sand was obtained from the manufacturer (M + E Tebbe-Neuenhaus GmbH & Co. KG, Bottrop-Kirchhellen, Germany).

Additionally, scanning electron microscopy (SEM) images of filter materials before (Fig. 1) and after the experiment (Fig. 4) were taken. Finally, the used filter materials were analyzed with the help of a confocal laser scanning microscope (cLSM) to reveal distribution pattern of the developed biofilm (Fig. 5) and for contamination with FIB (Fig. 3).

### Filter design

The experimental setup consisted of six glass filter columns with an inner diameter of 5 cm and a total height of 180 cm (Fig. 1). The filters were designed as AnBFs and contained a 60 cm filter bed which was supported by two 6 cm quartz-gravel layers (3–5 mm diameter at the bottom followed by a 3–4 mm diameter layer). The AnBFs were operated under saturated conditions. To prevent biochar material from floating, the top 3 cm of the biochar bed was filled in a mosquito net and weighted with a stainless steel nut. AnBFs were operated in triplicates and covered with aluminum foil to prevent algal growth. The minimum supernatant water level was adjusted by an overflow weir at 30 cm above the filter bed. Due to the development of a *schmutzdecke* (dirt layer) on the filter bed that causes a decrease in permeability over time, the supernatant water layer of all filters was allowed to rise about 78 cm to compensate the head loss in the filter bed. This ensured a constant water flow over the entire experiment. As soon as supernatant water of one filter reached the emergency overflow at the top of the filter column, the top 10 cm of all filters and the mosquito net pillow of biochar filters were removed and replaced.

### Operating parameters

The filters were operated with municipal wastewater that was received from the municipal full scale treatment plant Ölbachtal (Ruhrverband, Bochum, Germany, 320,000 PE; Table 2) for 70 days. Effluent from the grit chamber was stored in an 2 m³ opaque barrel for two days to simulate a wastewater pre-treatment by an anaerobic pond, which has a similar HRT^50^ and removed TSS and COD. Afterwards, the surface water layer (approximately 140 L) was decanted and stored in an opaque barrel, protected from light and cooled to 4–6 °C (T 2200, Lauda, Lauda-Königshofen, Germany) to prevent die-off of FIB and biological degradation during storage time. To avoid sedimentation, the storage reservoir was continuously stirred (IKA RW 10 R, IKA-Werke, Staufen, Germany) and renewed every two days.

**Table 2:** Mean values of raw wastewater, which was used in this experiment.

Parameter	Unit	Raw wastewater
COD	mg∙L^−1^	375 ± 158 (131)
NH_4_-N	mg∙L^−1^	42 ± 15 (131)
P_tot_	mg∙L^−1^	7 ± 3 (120)
TSS	mg∙L^−1^	212 ± 140 (131)

Standard deviations are presented as variation range and number of samples are given in brackets.

The filters were fed by peristaltic pumps (Watson Marlow 205 S and Watson Marlow 323 S; Falmouth, United Kingdom) with a constant HLR of 0.05 m∙h^−1^. The mean organic surface load was 366 ± 125 g_COD_∙m^−2^∙d^−1^ and 111 ± 37 g_TOC_∙m^−2^∙d^−1^, respectively. This resulted in a mean OLR of 509 ± 173 g_COD_∙m^−3^∙d^−1^ and 154 ± 52 g_TOC_∙m^−3^∙d^−1^ (Table 1). The experiment was performed at constant room temperature (22 ± 1 °C) with an empty bed contact time of 14.4 h.

### Sampling

**Figure 6:**
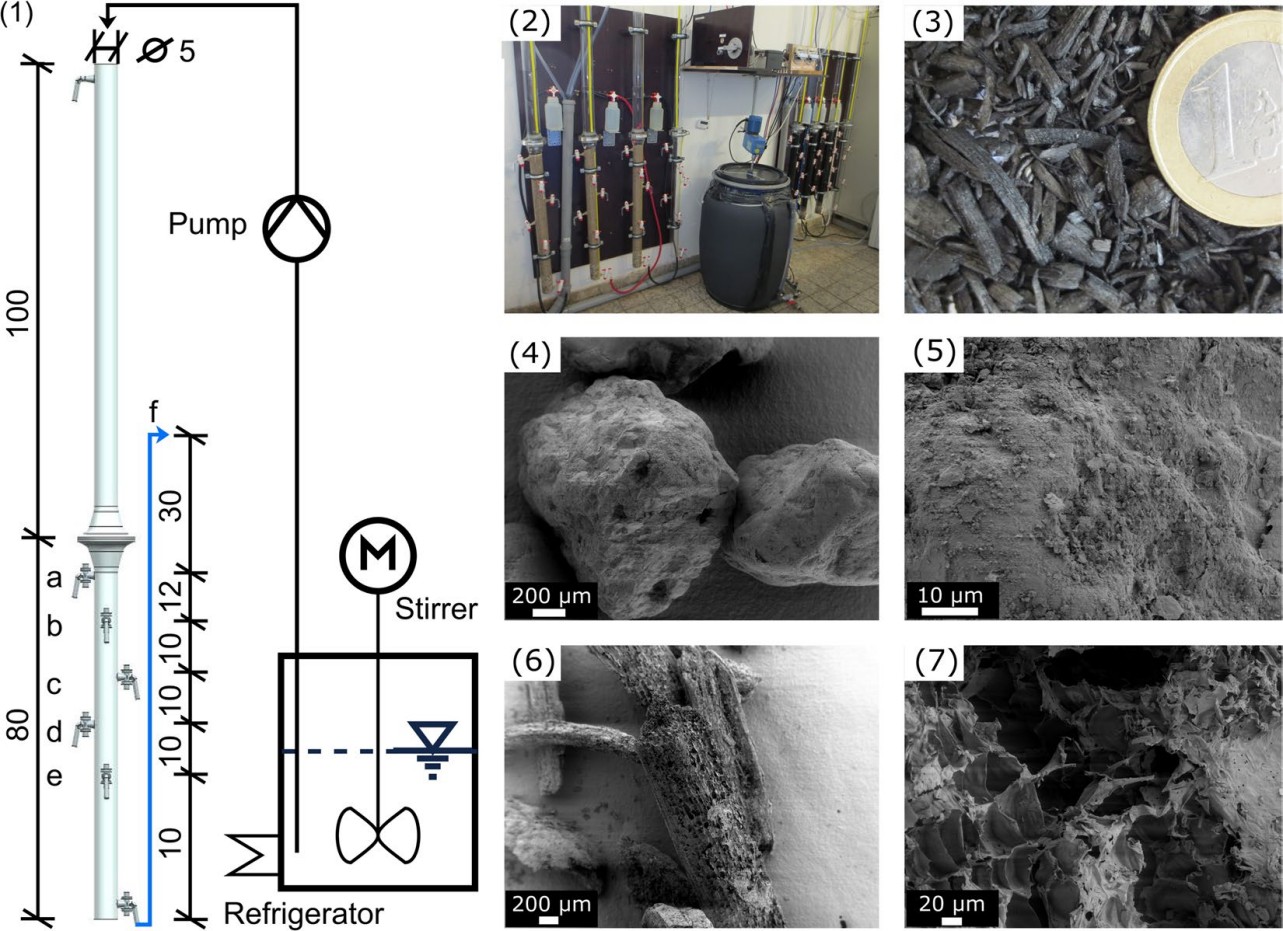
Schematic diagram of column design and filter setup (1). Letters a-e represent sampling ports. Overflow weir and filter effluent is marked as (f). The values are given in cm units. Picture of experimental setup (2) and miscanthus biochar (3). Scanning electron images of sand (4 and 5) and of biochar (6 and 7).

Before the first sampling filters were operated with wastewater for six days. Samples of influent and effluent were taken in sterile 50 mL tubes and tested immediately for FIB *E*. *coli* and enterococci, temperature, pH, EC and turbidity as well as stored for further physico-chemical analysis, namely NH_4_-N, P_tot_, COD and TOC at −20 °C. To develop a removal profile over filter depth, water samples were taken at the sampling ports (b), (c), (e) and the effluent (f, Fig. 6) 50 days after filter activation and analyzed for FIB concentrations.

After 70 days, one sand and one biochar filter have been dismantled to sample filter material at the filter depths 0–5 cm (direct below the *schmutzdecke*), 10–15 cm, 20–25 cm and 40–45 cm. Filter material was tested for FIB and prepared for scanning electron microscope (SEM) and confocal laser scanning microscope (cLSM) analysis.

### Microbial analysis and sample preparation

*E*. *coli* and enterococci were quantified using the standardized Most Probable Number (MPN) microplate methods for wastewater DIN EN ISO 9308-3 and DIN EN ISO 7899-1, respectively according to manufacture guidelines (Bio-Rad, Munich, Germany).

Filter material was analyzed for FIB according to previous studies^51^. Briefly, samples of the filter materials were transferred into sterilized 100 mL glass bottles pre-filled with 50 mL Dehydrated Special Microplates diluent (DSM; pH 7.4; Bio-Rad, Munich, Germany). Subsequently, the 100 mL glass bottles were placed in an ultrasonic bath for 20 min to remove the attached FIB from the filter materials. The resulting solution of the DSM and detached biofilm was used to determine the FIB concentration. After the removal of the FIB, the dry weight (DW) of the filter material was determined at 105 °C, and its FIB concentration reported as MPN∙cm^−3^, using the specific bulk density of the materials.

### Biofilm staining for cLSM microscope analysis

For the analysis of the biofilm on the different filter materials, the extracellular polymeric substances (EPS), especially the glycoprotein fraction, were marked with a lectin labelled with Alexa Fluor 488 dye. Alexa Fluor 488 labelling were done using a commercial protein labelling kit (Invitrogen/Thermo Fisher Scientific, MA USA) and *Aleuria aurantia* Lectin (Vector Laboratories, CA USA) according to the manufacturer´s instructions. After twofold washing with sterile tap water, filter material was gently fixed with silicone in a petri dish. The remaining water on the top of the filter material was carefully removed with a lint-free cloth. At first, the material was marked with about 50 µl Lectin-Alexa Fluor 488 (0.9 M) for 20 minutes in the dark at room temperature. After incubation the filter material was washed twice again with sterile tap water. The remaining water on the top of the filter material was carefully removed with a lint-free cloth. For analysis of total bacteria, the material was stained with SYTO60 (20 µM) for 20 minutes in the dark at room temperature. After incubation the petri dish was filled with sterile tap water so that the filter material was about 5 mm under water.

### Confocal laser scanning microscopy (cLSM) and z-stack analysis

A cLSM (TC SP8, Leica microsystems, Germany) was used for biofilm analysis on the filter materials. To compile three-dimensional images of the biofilm, image stacks were created with an image area of 465 × 465 µm (in x- and y-axis) and a distance of 2 µm (z-axis) between the images. Therefore, a 25-fold dipping lens (HCX IRAPO L25x/0.95 W, Leica microsystems, Germany) was used. The settings for the scans were listed in Table S2. Further parameters, such as gain and off-set, were selected for each shot to avoid over- or underexposure. The so generated z-stacks were analyzed with open source ImageJ software and the plugin ‘voxel counter’ to determine the percentage of the EPS and total bacteria of the created biofilm z-stacks. The voxel size of 0.455 × 0.455 × 2.0 µm was dependent of the chosen biofilm area and the slice distance (Table S2). The default settings of the software were used except the threshold. For the threshold the Huang algorithms was chosen, and manual adjusted.

### Physico-chemical parameter

COD was measured according to DIN 38409-H41-H44, using the cuvette tests LCK 314, LCK 514 or LCK 614 (Hach, Düsseldorf, Germany), depending on expected COD concentration. P_tot_ was measured with LCK 350 cuvette tests (Hach, Düsseldorf, Germany), according to DIN EN ISO 6878-1-1986 and NH_4_-N according to DIN 38406 E5-1, using LCK 303 cuvette tests. TOC was measured according to DIN EN1484 with a DIMATOC 2000 (DIMATEC, Essen, Germany).

Turbidity was measured according to DIN ISO 7027 with a Hach turbidimeter 2100 P ISO (Lange, Düsseldorf, Germany). TSS were determined according to the standard method DIN 38409-2. A Multi 3430 (WTW, Weilheim, Germany) was used for electrochemical analyses (pH, T, EC, and redox potential). A SenTix 980 probe was used for pH and temperature measurements, while a TetraCon 925 was used to determine the electrical conductivity and a SenTix ORP-T 900 probe for redox potential (WTW, Weilheim, Germany).

### Data analysis

For analysis of physico-chemical and microbial parameters, data of triplicates were pooled together. Data were tested for normal distribution using Shapiro-Wilk-test with a level of significance of *p* < 0.05. Significance of differences in the mean were analyzed using Student’s t-Test with *p* ≤ 0.05 as the threshold for significance. Correlation analyses were performed for the important parameters turbidity, *E*. *coli*, COD, TOC, P_tot_ and NH_4_-N, to identify relationships between their removal rates over experimental time. Pearson’s correlation coefficients as a measure of the strength of the association between the variables and corresponding *p*-values were calculated using Student’s t-Test as an indicator for the significance of the correlation coefficient for filter effluent concentrations (Fig. S7a,b), for normalized effluent concentrations (C⋅C_0_^−1^), dividing the effluent concentration (C) through influent concentration (C_0_; Fig. S7c,d), as well as for removal efficiencies of AnBFs (Fig. S7e,f). Statistical analyses were performed with OriginPro 2017 (Northampton, MA, USA).

## Supplementary information

Supplementary Information.
