# Supplementary material for: Slow sand filtration of raw wastewater using biochar as an alternative filtration media
Source: Sci Rep. 2020 Jan 27;10:1229. doi: 10.1038/s41598-020-57981-0 (PMC6985130; doi:10.1038/s41598-020-57981-0)
Supplement: Supplementary file 1 — Supplementary Information. [file 41598_2020_57981_MOESM1_ESM.pdf]

Supporting materials for

Slow sand filtration of raw wastewater using biochar as an alternative filtration media

Korbinian Kaetzl[*]*, Manfred Lübken, Edith Nettmann, Stefan Krimmler and Marc Wichern*

Institute of Urban Water Management and Environmental Engineering, Fakultät für Bau- und Umweltingenieurwissenschaften, Ruhr-Universität Bochum, Universitätsstr. 150, 44780 Bochum, Germany.

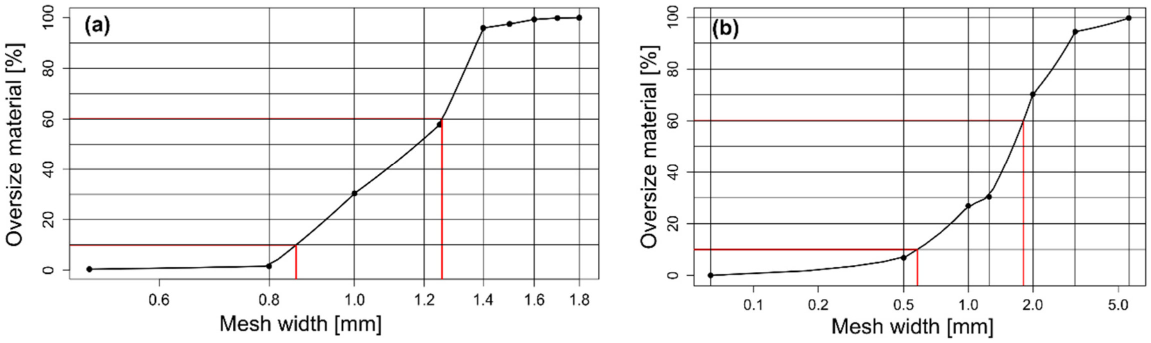

Figure S1. Grain size distribution of sand (a) and biochar (b).

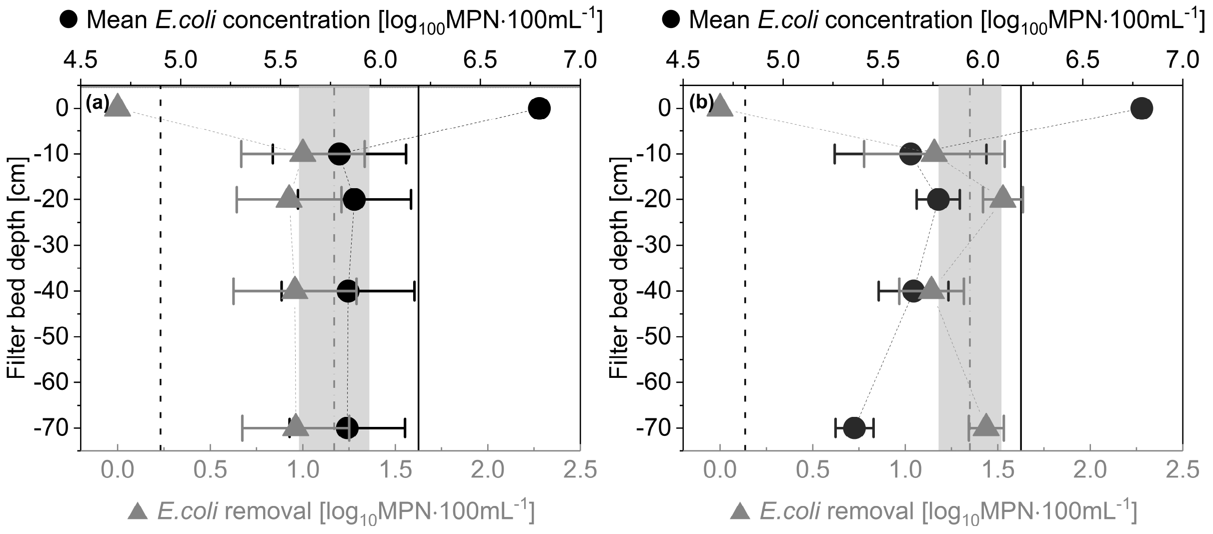

Figure S2. Mean *E. coli* concentration and removal over filter depth for sand (a) and biochar (b) filters (n = 3). 70 cm marks represent filter effluent and error bars represent standard deviation. Vertical grey dashed-dotted line represents mean *E. coli* removal rate over experimental time (n = 23) with 95 % confidence interval (grey area). Solid black line represents mean influent concentration and black dashed line the mean effluent concentration of *E. coli.*

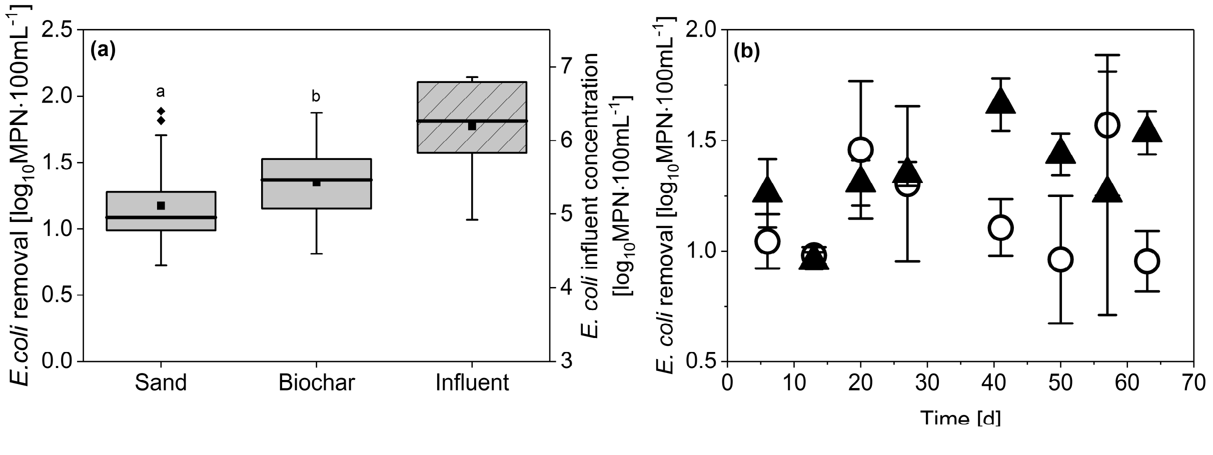

Figure S3. Boxplots of mean $\log_{10}$ removal of *E. coli* through sand and biochar filtration and influent concentration (a). Whiskers represent 1.5 interquartile range and mean values are presented as black squares. Letters indicate a significant difference of the mean (p <0.05) after post-hoc analysis. Mean removal rates of *E. coli* over experimental time for sand (circles) and biochar (black triangle) over experimental time (b). Error bars represent standard deviations.

Table S1. Properties of used filter materials. Specific surface area of sand was calculated according to Wichern et al.[1]

Parameter	Unit	Sand	Biochar
**Max. diameter (d_{max})**	mm	1.8	> 5.6
**Effective diameter (d_{10})**	mm	0.86	0.58
**d_{60}**	mm	1.26	1.81
**Uniformity coefficient (U)**		1.47	3.12
**Porosity**	%	40	59
**HRT_{min}***	h	12	14.8
**HRT_{max}****	h	27.6	30.4
**Bulk density**	g cm^{-3}	1.53	0.12
**Specific surface area (BET)**	m^2 g^{-1}	< 0.01	500
**Specific surface area (BET)**	m^2 m^{-3}	$< 1.53 \cdot 10^4$	$6.13 \cdot 10^7$
**Surface area (BET) per filter bed**	m^2	< 18	72,000

*with a minimum headwater level of 30 cm

**with a maximum headwater level of 102 cm

Table S*2*. Chemical characteristics of biochar, which was used in AnBF.

Parameter	Unit	Biochar
**Ash**	%	1.4
**C**	%	80.0
**N**	%	0.5
**O**	%	8.1
**H**	%	1.3
**H/C**	Molar ratio	0.19
**O/C**	Molar ratio	0.08
**P**	mg·kg^{-1}	2,686
**Al**	mg·kg^{-1}	654
**Fe**	mg·kg^{-1}	745
**K**		17,978
**Mg**	mg·kg^{-1}	2,350
**Na**	mg·kg^{-1}	886

Table S3. Chemical characteristics of sand, which was used in AnBF.

Parameter	Unit	Sand
**Na_2O**	% w·w^{-1}	0.09
**MgO**	% w·w^{-1}	0.02
**Al_2O_3**	% w·w^{-1}	1.3
**SiO_2**	% w·w^{-1}	97.6
**K_2O**	% w·w^{-1}	0.87
**CaO**	% w·w^{-1}	0.02
**TiO_2**	% w·w^{-1}	0.024
**Fe_2O_3**	% w·w^{-1}	0.067
**P_2O_5**	% w·w^{-1}	< 0.01

Table S4. cLSM settings

Parameters	Settings
**Extinction (Alexa Fluor 488; EPS)**	488 nm
**Emission (Alexa Fluor 488; EPS)**	490 – 550 nm
**Extinction (SYTO60; total cell count)**	633 nm
**Emission (SYTO60; total cell count)**	640 – 700 nm
**z-stack slice distance**	2 µm
**Picture area**	465 x 465 µm
**Scanner speed**	200 hz
**Picture resolution**	1024 x 1024 pixel
**Voxel size**	0.455 x 0.455 x 2.0 µm

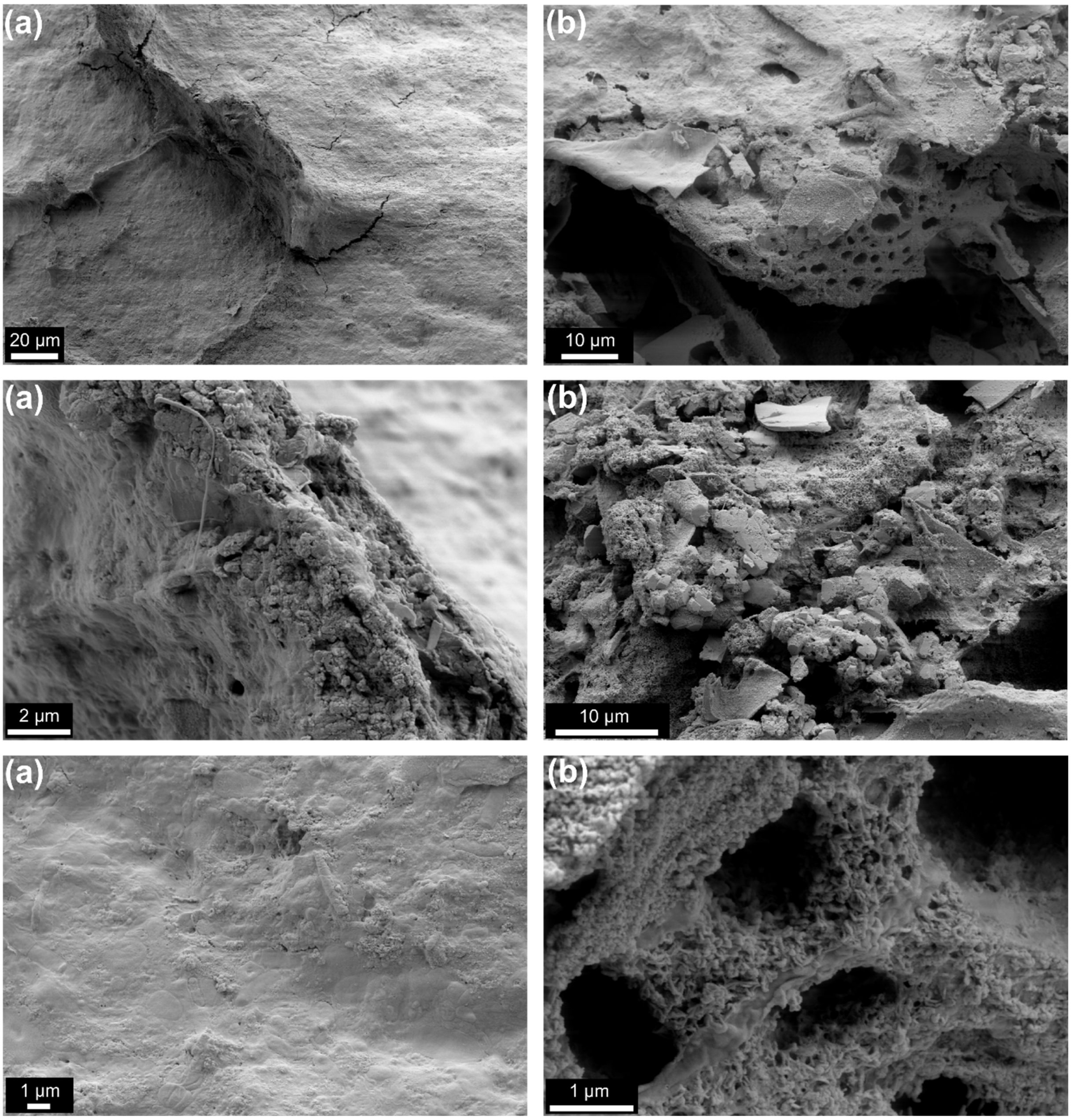

Figure S4. Scanning electron images of sand (a) and biochar (b) filter material after 70 days at 10 ± 2.5 cm.

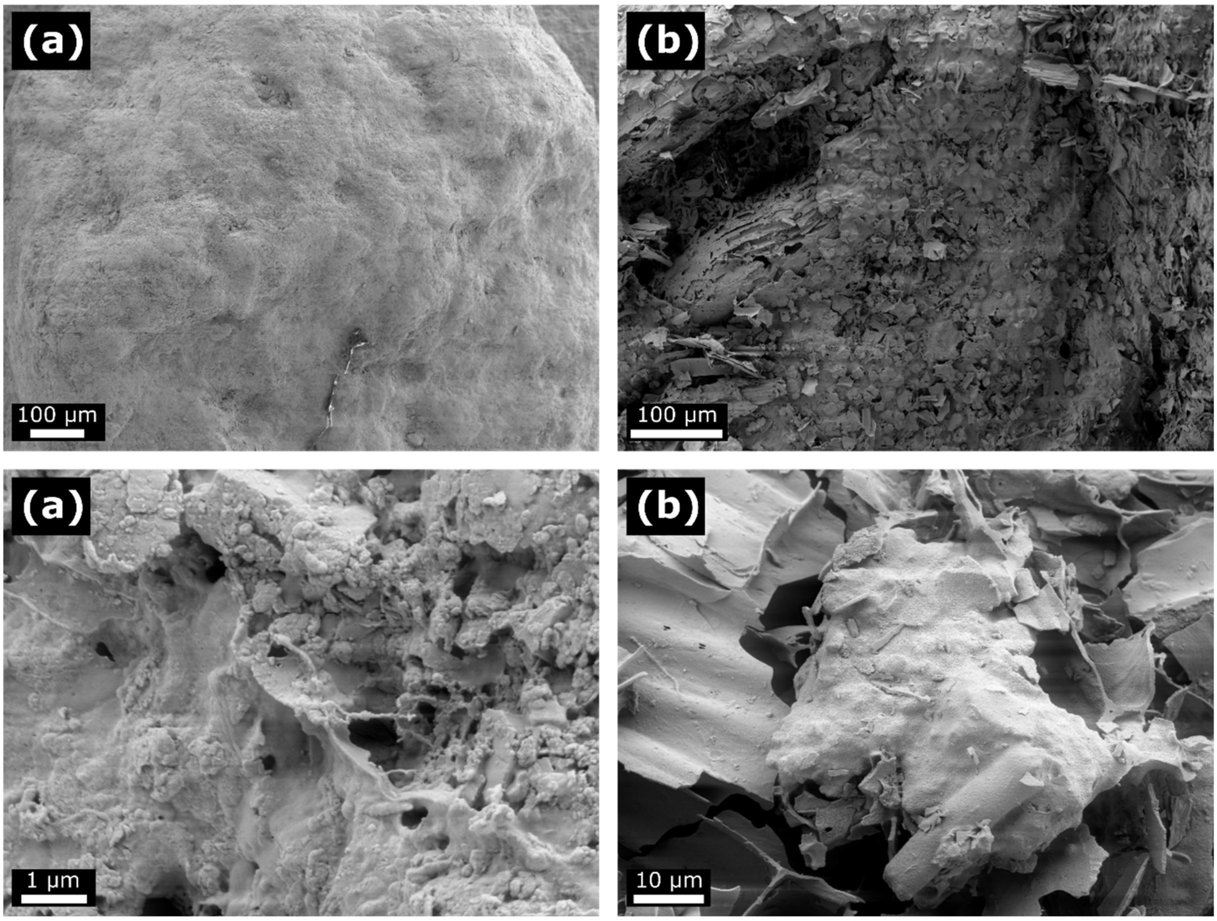

Figure S5. Scanning electron images of sand (a) and biochar (b) filter material after 70 days at 30 ± 2.5 cm.

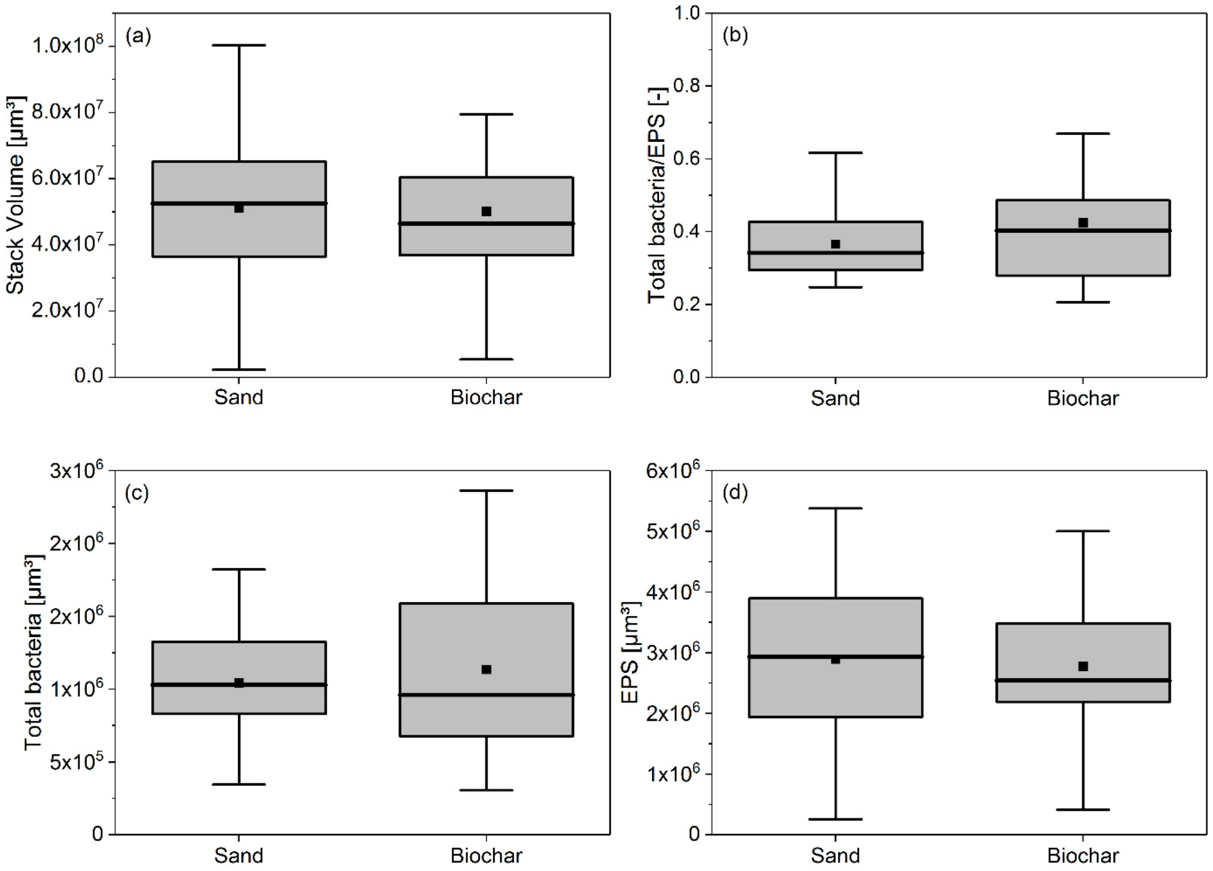

Figure S6. Results from cLSM microscope analysis of sand (n = 31) and biochar (n = 25) particles. Average stack volume (a) of scans, total bacteria/EPS ration (b), Volume of total bacteria (c) and Volume of EPS- Glycoprotein (d) .

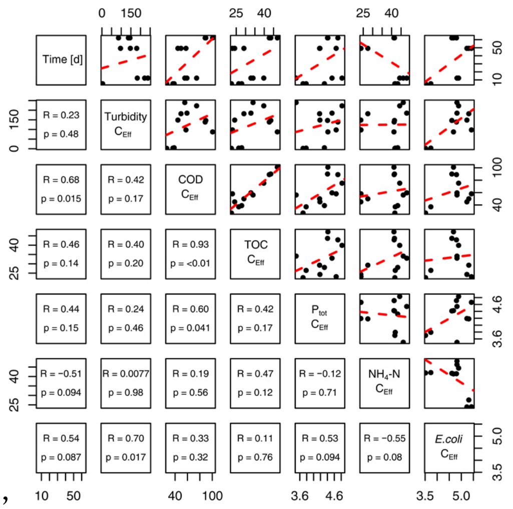

Figure S7a. Correlation analysis for effluent concentrations of biochar filters for Tubidity [FNU]; COD, TOC, P_{tot} and NH_4-N [$mg \cdot L^{-1}$] and *E. coli* [$log_{10}MPN \cdot 100mL^{-1}$] over time.

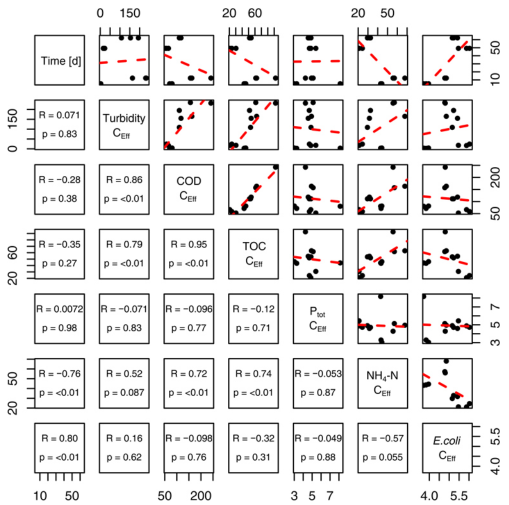

Figure S7b. Correlation analysis for effluent concentrations of sand filters for Tubidity [FNU]; COD, TOC, P_{tot} and NH_4-N [$mg \cdot L^{-1}$] and *E. coli* [$log_{10}MPN \cdot 100mL^{-1}$] over time.

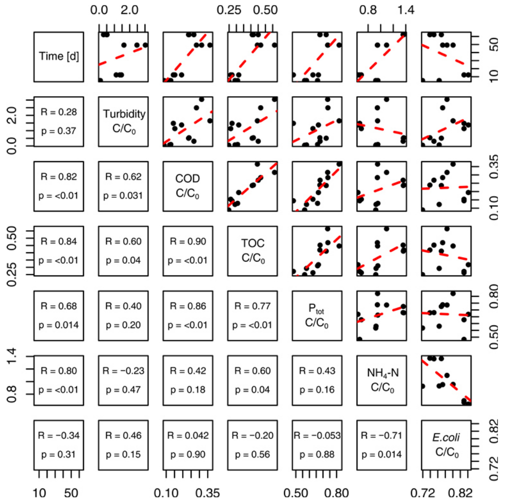

Figure S7c. Correlation analysis for normalized ($C \cdot C_0^{-1}$) concentrations of biochar filters, using influent (C_0) and effluent concentrations (C) of parameters over time.

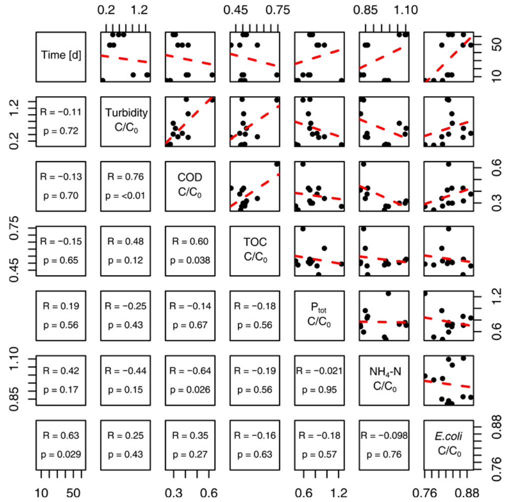

Figure 7d. Correlation analysis for normalized ($C \cdot C_0^{-1}$) concentrations of sand filters, using influent (C_0) and effluent concentrations (C) of parameters over time.

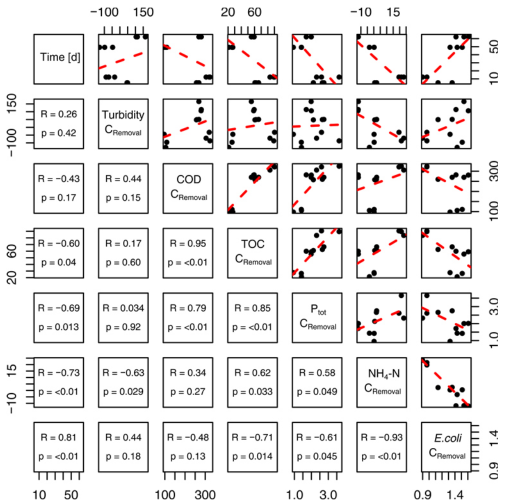

Figure S7e. Correlation analysis for removal efficiency of biochar filters for Tubidity [FNU]; COD, TOC, P_{tot} and NH_4-N [$mg \cdot L^{-1}$] and *E. coli* [$log_{10}MPN \cdot 100mL^{-1}$] over time.

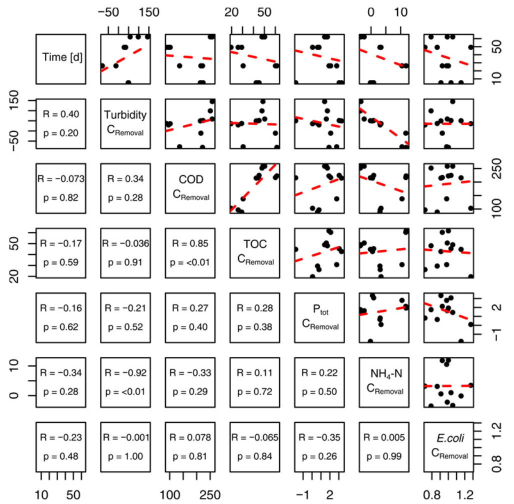

Figure S7f. Correlation analysis for removal efficiency of sand filters for Tubidity [FNU]; COD, TOC, P_{tot} and NH_4-N [$mg \cdot L^{-1}$] and *E. coli* [$log_{10}MPN \cdot 100mL^{-1}$] over time.

Figure S7. Correlation analysis between selected parameters over experimental time (n=12), for biochar (7a, 7c, 7e) and sand (7b, 7d, 7f) filter, using linear regression. The Pearson correlation coefficient between the individual parameters is represented as "R" with the corresponding *p*-value.

References

1. Wichern, M., Lindenblatt, C., Lübken, M. & Horn, H. Experimental results and mathematical modelling of an autotrophic and heterotrophic biofilm in a sand filter treating landfill leachate and municipal wastewater. *Water Res.* **42**, 3899–3909 (2008).
